# Joint reconstruction and classification of tumor cells and cell interactions in melanoma tissue sections with synthesized training data

**DOI:** 10.1007/s11548-019-01919-z

**Published:** 2019-02-16

**Authors:** Alexander Effland, Erich Kobler, Anne Brandenburg, Teresa Klatzer, Leonie Neuhäuser, Michael Hölzel, Jennifer Landsberg, Thomas Pock, Martin Rumpf

**Affiliations:** 1 Institute for Numerical Simulation, University of Bonn, Bonn, Germany; 2 Institute of Clinical Chemistry and Clinical Pharmacology, University of Bonn, Bonn, Germany; 3 Institute of Computer Graphics and Vision, Graz University of Technology, Graz, Austria; 4 Department of Dermatology and Allergy, University of Bonn, Bonn, Germany

**Keywords:** Image reconstruction and classification, Variational networks, Digital pathology, Tumor immune cell interaction, Nuclei detection, 68T45, 68T10

## Abstract

**Purpose:**

Cancers are almost always diagnosed by morphologic features in tissue sections. In this context, machine learning tools provide new opportunities to describe tumor immune cell interactions within the tumor microenvironment and thus provide phenotypic information that might be predictive for the response to immunotherapy.

**Methods:**

We develop a machine learning approach using variational networks for joint image denoising and classification of tissue sections for melanoma, which is an established model tumor for immuno-oncology research. The manual annotation of real training data would require substantial user interaction of experienced pathologists for each single training image, and the training of larger networks would rely on a very large number of such data sets with ground truth annotation. To overcome this bottleneck, we synthesize training data together with a proper tissue structure classification. To this end, a stochastic data generation process is used to mimic cell morphology, cell distribution and tissue architecture in the tumor microenvironment. Particular components of this tool are random placement and rotation of a large number of patches for presegmented cell nuclei, a stochastic fast marching approach to mimic the geometry of cells and texture generation based on a color covariance analysis of real data. Here, the generated training data reflect a large range of interaction patterns.

**Results:**

In several applications to histological tissue sections, we analyze the efficiency and accuracy of the proposed approach. As a result, depending on the scenario considered, almost all cells and nuclei which ought to be detected are actually marked as classified and hardly any misclassifications occur.

**Conclusions:**

The proposed method allows for a computer-aided screening of histological tissue sections utilizing variational networks with a particular emphasis on tumor immune cell interactions and on the robust cell nuclei classification.

## Introduction

Solid cancers are almost always diagnosed by histopathologists, mainly based on hematoxylin and eosin (H&E)-stained slides. The phenotypic information about nuclear atypia, cell morphology, cellular density and tissue architecture are essential characteristics for accurate diagnosis. Over the last decades, molecular and genomic approaches have revolutionized our understanding of tumor biology and are incorporated for diagnostic, prognostic and therapeutic purposes. The unexpected clinical success of immunotherapy by targeting the immune system using immune checkpoint inhibitors like anti-PD1- and/or anti-CTLA4 antibody in different tumor entities has proven the importance of tumor immune cell interactions within the tumor microenvironment [12]. However, the therapeutic efficacy is limited by primary and acquired resistance to immunotherapy. Thus, there is a growing need to identify predictive biomarkers and to enhance our understanding of the complex interactions between the immune system and tumor cells. Therefore, the evaluation of phenotypic information of tumor cells and the surrounding cells, including immune, vessel and stroma cells by detailed histopathological analyses, has become highly clinically relevant. Visual information embedded in histological tumor samples reflects the underlying molecular pathology. Quantitative imaging features can thus be expected to contain predictive power as biomarkers for cancer patients. However, the evaluation of histology is highly subjective and known for its inter- and intraobserver variations. Pathologists are also limited by scale and need to reduce information into categorical descriptions. Image analysis algorithms that precisely describe morphologic features provide tremendous opportunities for integration for genotype–phenotype comparison. Technological advances in digital pathology, imaging and computing are currently creating new tools for exploring relationships between morphology and molecular and genomic alterations in cancer tissues. Machine learning has emerged as an important image analysis tool that provides exciting opportunities to improve our understanding of cancer biology, immunology and ultimately patient care.

Melanoma is one of the most aggressive forms of skin cancer. Spontaneous melanoma regression, the identification of the first cancer antigen in melanoma and the high response rate to immunotherapy in melanoma patients have established melanoma as a model tumor for immuno-oncology research that increases our understanding of tumor immune cell interactions in other types of cancer. It has been shown that tumor-infiltrating CD8+ immune cells and their distribution within the tumor microenvironment are promising predictive biomarkers [16]. The presence of immune infiltrates and their spatial distribution in different cancer entities has recently been analyzed by using H&E images of the digital slide archive of The Cancer Genome Atlas (TCGA) and a convolutional neural network that classified $50\mu m$-square image patches for TIL content [14]. In [17], Turkki et al. exploited a combination of pretrained convolutional neural networks and support vector machines to detect immune cell-rich areas in H&E-stained histological sections of breast tissues. We could recently show [4] that direct interactions of immune cells with melanoma cells can be detected by a deep learning approach using variational networks for joint image reconstruction and segmentation.

Despite the successful use of deep learning approaches in tumor immunology, intratumoral heterogeneity as a tumor biopsy often contains a range of histological patterns and thus represents a significant challenge that might be overcome by a large amount of labeled data. For example in [15], Sirinukunwattana et al. employed a spatially constrained convolutional neural network for the detection of relevant cell nuclei using a neighboring ensemble predictor to incorporate the spatial structure of the cells. In [18], a classification of epithelial and stromal cells based on a deep convolutional neural network is performed, where the quality of the outcome is compared to handcrafted features such as local binary patterns. Liu et al. [10] proposed a learning-based automatic segmentation algorithm relying on seed detection and contour refinement to quantify morphologic characteristics of muscle fibers for H&E-stained skeletal muscle sections. For a recent overview of deep learning methods for classification and segmentation tasks in digital pathology, we refer to [6].

Here, we pick up the variational networks from image reconstruction presented in [8] to learn a coupled reconstruction and segmentation approach for the automatic detection and classification of cell markers in the context of computer-aided cancer diagnosis. We avoid the manual annotation of real training data, which would require substantial user interaction of experienced pathologists for each single training image, by extracting structural, color and noise information from just a few real histological sections of cancer tissues. These bits of information are used to generate training images that mimic real histological sections using a stochastic cell distribution algorithm. Particular components of this tool are random placement and rotation of a large number of patches for presegmented cell nuclei, a stochastic fast marching approach to mimic the geometry of cells and texture generation based on a color covariance analysis of real data. Here, the generated training data reflect a large range of interaction patterns. We synthesize data to train the parameters of an iteratively evolving variational network. Experiments indicate that the thus trained networks generalize to real histological sections.

In this paper, we extend the preliminary work presented in [4] by an improved design of the variational network with an effective direct coupling of the image and the segmentation mask channel. In addition, we incorporate a stochastic data generation process used to mimic cell morphology, cell distribution and tissue architecture in the tumor microenvironment. Furthermore, we discuss in detail the layer architecture of the training data generator. Finally, we investigate three different classification tasks.

The paper is organized as follows: in Sect. 2, the extension of variational networks with the direct coupling of different channels is derived. Then, three algorithmic challenges related to immunofluorescently and H&E-stained histological sections are briefly presented, and the data acquisition procedure is described in Sect. 3. In Sect. 4, the stochastic training data generation process is presented. Finally, the applicability and accuracy of the proposed methods are validated for various applications to real image data in Sect. 5.

## A coupled variational network

We consider a pair of a noisy and blurry $N_1\times N_2$ color image $\mathbf {u}_0\in \mathscr {U}=([0,1]^3)^{N_1\times N_2}$ and an initial segmentation mask $m_0 \in \mathscr {M}=[0,1]^{N_1\times N_2}$, where the corresponding binary mask is computed via thresholding at the value $\frac{1}{2}$. Our aim is to reconstruct a clean image $\mathbf {u}\in \mathscr {U}$ and to compute a mask $m\in \mathscr {M}$ segmenting the task-dependent cell types. To tackle this joint reconstruction and segmentation task, we “deepen” the variational network [8] structure to handle coupled variables. The proposed variational network performs $N_t$ projected gradient steps of the form

1 $$\begin{aligned} \left\{ \begin{array}{ll} \mathbf {u}_{t+1}&{}=\text {proj}_{\mathscr {U}}(\mathbf {u}_{t}-\nabla _{\mathbf {u}} E_t(\mathbf {u}_{t}, m_{t})) \\ m_{t+1}&{}=\text {proj}_{\mathscr {M}}(m_{t}-\nabla _{m} E_t(\mathbf {u}_{t}, m_{t})) \end{array} \right. \quad \text {for }t\in \{0,\ldots ,N_t-1\}, \end{aligned}$$

where the operators $\text {proj}_{\mathscr {U}}$ and $\text {proj}_{\mathscr {M}}$ denote a pointwise projection on the sets $\mathscr {U}$ and $\mathscr {M}$, respectively. Note that this scheme can be interpreted as a discretized reaction–diffusion equation (cf. [2]) or as the time-discrete projected gradient flow [8] of a time-dependent variational energy. In this work, we propose to use the following coupled variational energy:

2 $$\begin{aligned} E_t(\mathbf {u}, m)&=\sum _{\mathscr {F}}\phi _t\left( K_t \left( \phi _t^\mathbf {u}(K_t^\mathbf {u}\mathbf {u}), \phi _t^m(K_t^mm)\right) \right) \nonumber \\&\quad +\tfrac{\lambda _t}{2}\Vert \mathbf {u}-\mathbf {u}_0\Vert _2^2. \end{aligned}$$

The first term couples the reconstructed image and the segmentation mask by means of convolution operations and parameterized nonlinear functions. The convolution operator

$$\begin{aligned} K_t^\mathbf {u} = (K_t^{\mathbf {u},1}, \ldots , K_t^{\mathbf {u},N_{f_\mathbf {u}}}) : \mathscr {U} \rightarrow \mathscr {F}_\mathbf {u} \end{aligned}$$

implements $N_{f_\mathbf {u}}$ 2D convolution kernels to extract features in $\mathscr {F}_\mathbf {u} = \mathbb {R}^{N_1\times N_2 \times N_{f_\mathbf {u}}}$ from the RGB image. Each of the $N_{f_\mathbf {u}}$ features is transformed by applying a distinct nonlinear pointwise function, which are then aggregated in

$$\begin{aligned} \phi _t^\mathbf {u} : \mathscr {F}_\mathbf {u} \rightarrow \mathscr {F}_\mathbf {u}. \end{aligned}$$

In the same fashion, the 2D convolution operator

$$\begin{aligned} K_t^m = (K_t^{m,1}, \ldots , K_t^{m,N_{f_m}}) : \mathscr {M} \rightarrow \mathscr {F}_m \end{aligned}$$

identifies and extracts features from the segmentation mask that are then nonlinearly transformed by the functions

$$\begin{aligned} \phi _t^m : \mathscr {F}_m \rightarrow \mathscr {F}_m. \end{aligned}$$

The resulting feature spaces $\mathscr {F}_\mathbf {u}$ and $\mathscr {F}_m$ are concatenated into a coupled feature space $\mathscr {F} = \mathbb {R}^{N_1\times N_2 \times N_f}$ with $N_f = N_{f_\mathbf {u}} + N_{f_m}$ convolution kernels. We use the notation $(\cdot ,\cdot )$ to indicate a concatenation. In a next step, these features are combined by using $N_f$ 2D convolution kernels

$$\begin{aligned} K_t = (K_t^{1}, \ldots , K_t^{N_f}) : \mathscr {F} \rightarrow \mathscr {F} \end{aligned}$$

followed by a third set of learned nonlinear functions

$$\begin{aligned} \phi _t:\mathscr {F} \rightarrow \mathscr {F} \end{aligned}$$

that are also applied in a pointwise fashion to each feature. The energy of the smoothness or regularization term is then given by summing up all resulting feature elements. The second term in (2) enforces data consistency between the RGB image $\mathbf {u}$ and the initial image $\mathbf {u}_0$ using the squared $\ell _2$-norm.

To apply this variational model to our iterative scheme (1), we need the gradients of the energy (2) with respect to both input variables. By applying the chain rule, we get

$$\begin{aligned} \nabla _{\mathbf {u}} E_t(\mathbf {u}, m)&= (K_t^\mathbf {u})^\top D\phi _t^{\mathbf {u}}(K_t^\mathbf {u}\mathbf {u})P_\mathbf {u} K_t^\top D\phi _t\left( K_t \left( \phi _t^\mathbf {u}(K_t^\mathbf {u}\mathbf {u}),\right. \right. \\&\quad \left. \phi _t^m\left( K_t^mm\right) \right) +\lambda _t(\mathbf {u}-\mathbf {u}_0),\\ \nabla _{m} E_t(\mathbf {u}, m)&= (K_t^m)^\top D\phi _t^{m}(K_t^mm)P_m K_t^\top D\phi _t\left( K_t \left( \phi _t^\mathbf {u}(K_t^\mathbf {u}\mathbf {u}),\right. \right. \\&\quad \left. \left. \phi _t^m(K_t^mm)\right) \right) , \end{aligned}$$

where the operators $P_\mathbf {u}$ and $P_m$ extract the parts of $\mathscr {F}$ that originate from $\mathscr {F}_\mathbf {u}$ and $\mathscr {F}_m$, respectively. Here, $D\phi _t^{\mathbf {u}}$, $D\phi _t^{m}$ and $D\phi _t$ denote the derivatives of the corresponding nonlinear functions. As in the variational networks [8], the derivatives $D\phi _t$ are parameterized using Gaussian radial basis functions with weights $w_t \in \mathbb {R}^{N_f \times N_w}$, where $N_w$ defines the number of radial basis functions. For the two feature transforming functions $\phi _t^\mathbf {u}$ and $\phi _t^m$, we have to evaluate both the functions and their derivatives. Thus, we parameterize the functions $\phi _t^\mathbf {u}$ and $\phi _t^m$ directly using Gaussian radial basis functions with weights $w_t^\mathbf {u} \in \mathbb {R}^{N_{f_\mathbf {u}}\times N_w}$ and $w_t^m \in \mathbb {R}^{N_{f_m} \times N_w}$. The derivatives can then be evaluated by differentiating the Gaussian basis functions. Using this formulation, we can group all trainable parameters of the scheme into $\theta = (K_t^\mathbf {u}, w_t^\mathbf {u}, K_t^m, w_t^m, K_t, w_t, \lambda _t)_{t=1}^{N_t}$. The input data consist of a RGB tissue section $\mathbf {u}_0$ as well as an empty segmentation mask $m_0$.

We solely incorporate a synthesized set of training images $(\mathbf {u}^s_0, m^s_0, \mathbf {g}^s_\mathbf {u}, g^s_m)_{s=1}^{N_s}$ to train the parameters $\theta$ of the entire scheme by minimizing the loss function

3 $$\begin{aligned} \underset{\theta \in {\mathscr {P}}}{\min } \, \sum _{s=1}^{N_s} \frac{1}{6} \Vert \mathbf {u}^s_T - \mathbf {g}^s_\mathbf {u} \Vert _2^2 + \frac{1}{2} \Vert m^s_T - g^s_m \Vert _2^2. \end{aligned}$$

Here, the target RGB image is denoted by $\mathbf {g}_\mathbf {u} \in \mathscr {U}$ and the target segmentation mask by $g_m \in \mathscr {M}$, respectively. The parameters are constrained to the set of admissible model parameters

$$\begin{aligned} {\mathscr {P}}&= \{ (K_t^\mathbf {u}, w_t^\mathbf {u}, K_t^m, w_t^m, K_t, w_t, \lambda _t) : K_t^{\mathbf {u},i}, K_t^{m,j} \in \mathscr {K}, \, \\&\qquad \Vert K_t^{l}\Vert _1 \le 1, \, \lambda _t \ge 0, \\&\qquad t = 1\ldots T, \, i = 1\ldots N_{f_\mathbf {u}}, \, \\&\qquad j = 1\ldots N_{f_m}, \, l = 1\ldots N_f \} , \end{aligned}$$

where the set $\mathscr {K} = \{K :\langle 1, K_t^{\mathbf {u},i} \rangle = 0, \, \Vert K_t^{\mathbf {u},i}\Vert _1 \le 1 \}$ refers to the 2D convolution kernels that have zero mean and lie in the $\ell _1$-norm ball. We use the $\ell _1$-norm to promote sparsity of the learned convolution kernels and thus increase the robustness of the model. Moreover, the kernels are required to have zero mean in order to improve their capability to extract textural information from the RGB image and denoise the segmentation mask. Note that we only bound the norm of the kernels $K_t$ in order to enable information exchange between the two feature spaces $\mathscr {F}_\mathbf {u}$ and $\mathscr {F}_m$. We scale the RGB term of the loss by a factor of $\frac{1}{6}$ to account for the larger number of channels. For learning, we use the Adam algorithm [7]. In each step of the Adam algorithm, we perform a projection of the parameters $\theta$ onto ${\mathscr {P}}$. The positivity constraint of the data term weights $\lambda _t$ is enforced by a truncation, and we use the Euclidean projection onto the $\ell _1$-ball as described in [3] for the filter kernels $K_t$. Finally, the feature extraction filters in $K_t^\mathbf {u}$ and $K_t^m$ are projected onto the set $\mathscr {K}$ using an accelerated gradient method, to account for both constraints simultaneously. The projection typically requires 4 to 20 iterations to converge. Moreover, the projection is computed in parallel for all 2D convolution kernels.

## Cell and nuclei classification tasks for melanoma tissue sections

In what follows, we will elaborate on three different classification tasks related to stained melanoma section images. More precisely, we focus on the detection of cells or cell nuclei encoded by biomarkers, where the spatial arrangement of cells indicating cell interactions is incorporated in some scenarios.

*Scenario 1* As direct tumor immune cell interactions are important for anti-tumor immunity, we establish as a first scenario a classification to identify immune cells in the proximity of tumor cells in melanoma section images with an immunofluorescence staining. CD45 positive immune cells are marked in red, cell nuclei are stained in blue by DAPI that binds to DNA, and melanocytes are stained for the melanocytic protein marker gp100 in green. Here, an immune cell is classified if the tumor cell concentration in a circular neighborhood with radius 40 pixels exceeds the threshold value 0.3. The values of all underlying pixels of classified immune cells are set to 1 in the ground truth marking channel of the synthesized training images, and all remaining pixels are set to 0.

*Scenario 2* To predict response to immunotherapy, melanoma tissue samples are usually classified into immune cell-rich and immune cell-poor tumors [1, 5]. Therefore, we detect immune cell-rich tumor areas in H&E-stained melanoma sections. Here, an immune cell is marked as classified if the concentration of immune cells in a circular neighborhood of radius 40 pixels is above the threshold value 0.2. A pixel in the ground truth mask is set to 1 if and only if it is located in the interior of classified immune cells and to 0 otherwise.

*Scenario 3* Finally, we focus on the detection of tumor cell nuclei in H&E-stained melanoma section images neglecting any cell interactions. However, the particular challenge of this approach arises from the similarity in the texture of tumor cell nuclei and the tumor microenvironment.

**Fig. 1:**
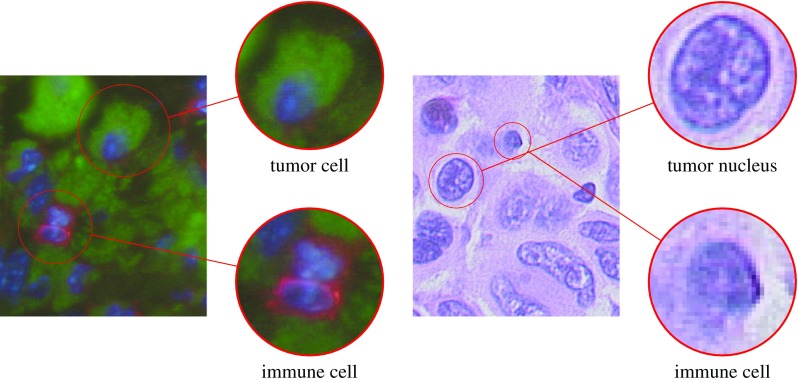
Melanoma tissue sections with immunofluorescence (left) and H&E (right) staining are shown. The upper magnifying glasses depict a tumor cell (left) and a tumor nucleus (right), in the lower magnifying glasses immune cells can be observed

In Fig. 1, exemplary melanoma tissue section images with an immunofluorescence (left) and a H&E (right) staining are depicted. The upper magnifying glasses point to a tumor cell (left) and a tumor nucleus (right), both lower magnifying glasses enlarge immune cells.

H&E-stained tissue images frequently exhibit intensity variations and deviations with regard to the mean color of cells of the same category. Thus, to enforce a comparable staining a normalization proposed by Reinhard et al. [11] is applied which amounts to a color transfer of a fixed reference image to all other images considered for Scenario 2 and Scenario 3.

### Acquisition of histological sections

Formalin-fixed paraffin-embedded (FFPE) murine melanomas (as previously described in [9]) as well as formalin-fixed paraffin-embedded and H&E-stained human melanoma samples of the Skin Cancer Center of the University Hospital in Bonn were used in this paper. The human samples were anonymized, and all patient-related data and unique identifiers were removed. H&E stains were performed according to standard protocols. Melanomas were immunofluorescently stained such that the immune cells are colored in red by the marker CD45, the tumor cells in green by the melanocytic marker gp100 and the nuclei in blue using the DAPI marker. Representative regions of interest containing primarily tumor nuclei and tumor-infiltrating immune cells were manually identified for each slide to prevent images containing tissue-processing artifacts, including bubbles, sections folds and poor staining. Stained sections were examined with a Leica DMBL immunofluorescence microscope. Regions of interest were cropped at $20\times$objective magnifications. All images were acquired with a JVC digital camera KY-75FU.

## Stochastic generation of training data

In this paper, all training images are synthesized by a stochastic data generation process mimicking structure, color distribution and noise of real histological sections of cancer tissues, where the generator is adapted for each aforementioned scenario. The major benefit of this approach lies in the unlimited availability of training data with an exact ground truth image and ground truth mask and thus circumvents the need for user interaction of an experienced pathologist.

The main guidelines of the stochastic data generation are as follows:

*Scripting language* All essential image features in each scenario such as the shape, the placement or the coloring of all object categories (cells, nuclei, connective tissue structures and background) are specified in a script which is then parsed in the configuration phase of the stochastic data generator.

*Layer concept* The training images with resolution $300\times 300$ are composed of a fixed number of layers, where each layer determines at most one object category. In the final step of the data generation, all layers are merged in reverse order in such a way that the data on the top layers replace any data it overlaps on the lower layers and the intersection of all transparent areas in the merged layers remains transparent, where transparency is encoded as usual in an additional alpha channel. The pixel values of the alpha channel of a specific layer are exactly 1 at all underlying pixels of objects in the layer. This procedure allows for a versatile and efficient description of the training data since image filters can be applied to each layer separately and placement rules for cell and tissue structures in different layers can easily be enforced. In particular, overlapping of cells or the location of cell midpoints inside other cells can be excluded for certain cell types.

*Ground truth segmentation mask* In all scenarios considered, the ground truth segmentation mask is derived from the cell structure in the layers according to task-dependent rules.

*Domain decomposition* The cells, nuclei and tissue structures are either placed randomly or in regions induced by a precomputed random Voronoi tessellation or a fast marching region decomposition with an underlying random velocity field drawn from a Gaussian distribution. The control points of both latter approaches are again chosen randomly.

*Geometry and texture* To mimic the geometry and the texture of the cells and the nuclei, we proceed in different problem-specific ways.

Apart from the tumor cells and associated nuclei in Scenario 3, all cells and nuclei are synthesized as ellipses, where both the length of the semi-axes and the inclination are drawn from a uniform distribution. To model the tumor nuclei in Scenario 3, a sample of tumor nuclei is cut out of real images and the patches thus obtained are placed stochastically on the layer with a random rotation. The shapes of the associated cells are again generated by a fast marching method with a fixed number of iterations, which is governed by an underlying stochastic velocity field and incorporates the patches as the initial mask.

Moreover, the mean color and the noise distribution of all cells and nuclei except for immune and tumor cells in Scenario 3 are drawn from a multivariate normal distribution, where the expectation, the variance and the covariance matrices of the noise are estimated from a small sample of patches of real images. We highlight that the aforementioned normalization of the H&E-stained images is essential for this extraction technique.

The texture of immune and tumor cells in Scenario 3 is composed of randomly chosen quadratic patches of fixed size in a lattice structure, where the patches are extracted from cells of the same type in real images. More precisely, due to the variability in the texture of both cell types, one additionally groups the patches into three cohorts depending on the mean color and one exclusively incorporates one cohort in the simulation of a specific cell to avoid heterogeneity artifacts. Furthermore, adjacent patches are aligned in a way that a moderate overlap is ensured and a blending in all overlap regions is performed to smoothen patch transitions.

*Connective tissue* The ribbon-type connective tissue in Scenario 2 and Scenario 3 that borders some of the regions specified above is modeled as the overlap of the preimage set of random intervals of several distance maps induced by a fast marching algorithm with different stochastic velocity fields, where the complement set of the region considered is the initial mask for the algorithm. The thickness of the tissue structures correlates with the size of the interval.

*Blood vessels and background* Finally, the blood vessels and the background are modeled as noise with average color, variance and covariance again extracted from samples of real images, where the blood vessels are restricted to certain regions.

We remark that the aforementioned various approaches to model the different cell types are necessitated by the specific requirements of each scenario. For example, since the immune cells, the tumor cells and the tumor nuclei in Scenario 3 are hardly distinguishable, a more involved modeling compared to the remaining scenarios is needed to capture finer image structures.

The task-dependent parameters and placement rules are listed in Table 1.

**Fig. 2:**
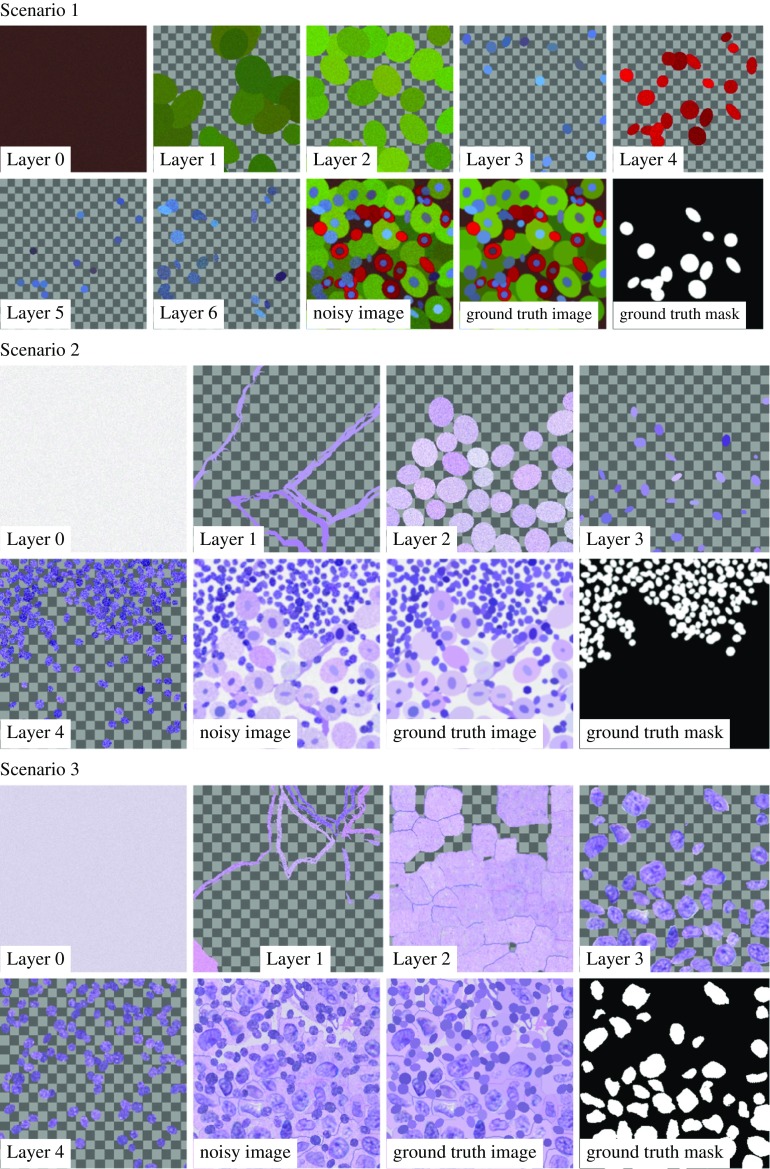
All layers that compose the noisy training images, the ground truth images and masks for Scenario 1 (first and second row), Scenario 2 (third and fourth row) and Scenario 3 (fifth and sixth row) are depicted

**Table 1:** Cell-specific data of all scenarios

	Category	Mean color	Semi-axes (pixels)	Number/placement
(1)	Tumor cell	Green	$20{-}35$	25 cells, random placement
	Tumor nucleus	Light blue	$8{-}12$	Only $60\%$ visible
	Immune cell	Red	$8{-}20$	20 cells, random placement, no overlapping
	Immune cell nucleus	Blue	$7{-}8$	Only $60\%$ visible
	Stroma/dediff. melanoma	Blue	$5{-}15$	20 cells, random placement, no overlapping
(2)	Tumor cell	Light purple	$15{-}25$	5 of 7 Voronoi regions filled, no overlapping
	Tumor nucleus	Purple	$3{-}10$	Only $60\%$ visible
	Immune cell	Violet	$4{-}8$	2 of 7 Voronoi regions filled, no overlapping
(3)	Blood vessel	Light red	–	1 of 11 fast marching regions filled
	Tumor nucleus	Patches	–	10 of 11 fast marching regions filled, no overlapping
	Tumor cells	Composition of patches	–	Fast marching with tumor nuclei as initial mask, three cohorts (each 55 patches)
	Immune cell	Composition of patches	$5{-}10$	Random placement, moderate overlap with tumor and immune cells allowed, three cohorts (each 60 patches)

Figure 2 depicts all layers that compose the training images, the resulting noisy training images obtained by merging the layers as well as the ground truth RGB image and the ground truth masks of all scenarios. Here, the checkerboard pattern encodes the alpha channel.

In Scenario 1, the background Layer 0 is composed of Gaussian noise incorporating the covariance analysis discussed above. The additional Layer 1 models partially hidden cell structures in the background. The top layers contain tumor cells (Layer 2) and immune cells (Layer 4) and their associated nuclei (Layer 3 and Layer 5) as well as stromal cells (Layer 6). Again, the mean color and the noise structure are extracted from samples of real images.

In Scenario 2, the background Layer 0 models the background noise, the foreground layers contain the connective tissue structures (Layer 1), tumor cells (Layer 2), visible tumor cell nuclei (Layer 3) and the immune cells (Layer 4), where immune cells in immune cell-rich areas enter into the ground truth mask.

Finally, in Scenario 3 the background noise structure is reflected by the Gaussian noise in Layer 0, and the blood vessels and the connective tissue in Layer 1 only occupy a small region of the image. The top layers contain the fast marching driven tumor cell structures along with cell contours (Layer 2), the patch-based tumor nuclei (Layer 3) and the immune cells (Layer 4). The ground truth mask depicts the visible part of the tumor nuclei, i.e., all pixels of the tumor nuclei that are not covered by immune cells.

**Fig. 3:**
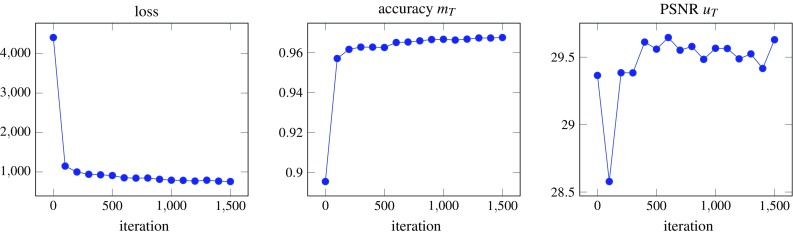
Evaluation of the loss function (left), accuracy of the segmentation $m_T$ (center) and the PSNR of the reconstructed PSNR image (right) for a training batch of Scenario 1

## Results

To train the proposed reconstruction and segmentation model (1), we use 800 stochastically generated training images of size $300\times 300$ for each scenario. The model outputs are generated by performing $N_t = 10$ parameterized steps. In each step, $N_{f_\mathbf {u}} = 20$ features are extracted from the RGB image using $11\times 11$ convolution kernels, while merely $N_{f_m} = 4$ kernels of size $5\times 5$ extract local information from the current segmentation. The zero-mean constraint for the RGB convolution kernels $K_t^\mathbf {u}$ is discarded for Scenario 1 which is motivated by an increased stability in the numerical experiments. After transforming the extracted features using the nonlinear functions, $N_t = 24$ convolution kernels of size $5\times 5$ are used to combine the RGB and segmentation channels. We parameterize all nonlinear functions with $N_w = 31$ Gaussian radial basis function that are equally distributed in the interval $[-1.2, 1.2]$. All parameters are trained applying 1500 iterations of the Adam optimizer with step sizes $10^{-4}$ (Scenario 1 and Scenario 2) and $5\cdot 10^{-5}$ (Scenario 3) and exponential decay rates $\beta _1=0.9$ and $\beta _2=0.999$ on a batch of size 8.

The trends of the loss function (3), the prediction accuracy of the segmentation mask $m_T$ and the peak signal-to-noise ratio (PSNR) of the reconstructed image $u_T$ are depicted in Fig. 3 for the training of Scenario 1. Initially, the segmentation accuracy is rather high (around $90\%$) which accommodates the structure of the target mask $g_m^s$ dominated by black and thus unsegmented pixels. Then, the accuracy increases consistently, whereas the PSNR values of the reconstructed image improve rather slowly.

**Fig. 4:**
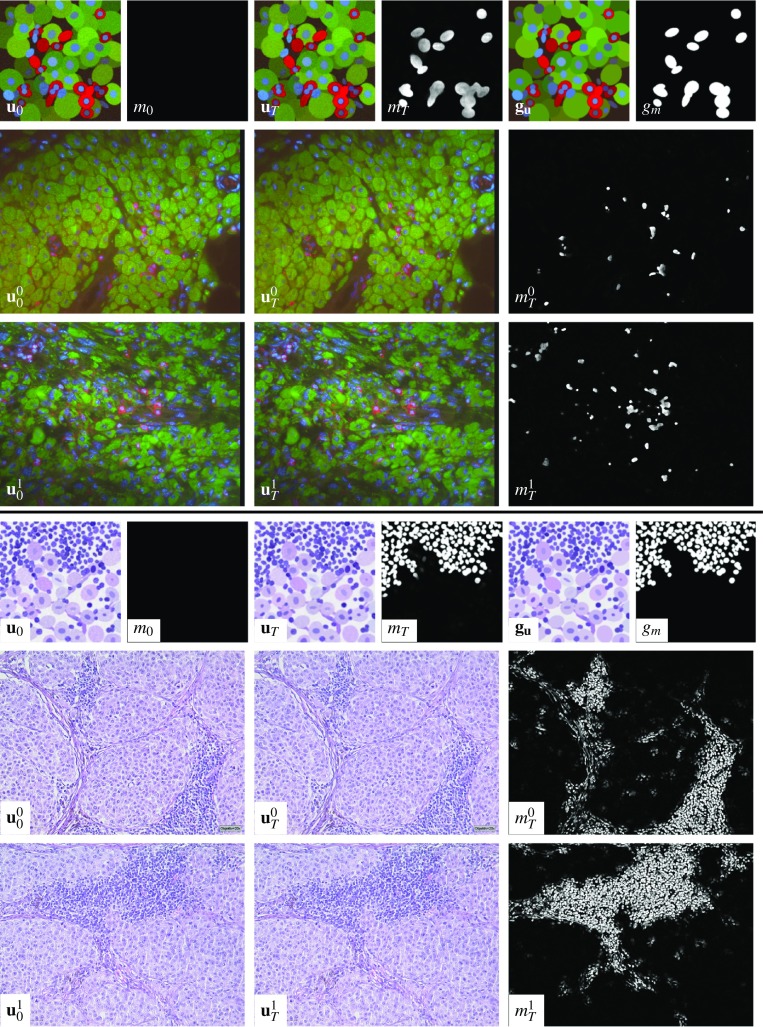
Training data (pairs of image and initial mask, output and ground truth) for Scenario 1 (first row) and Scenario 2 (fourth row). $\mathbf {u}^{i}_0$, $\mathbf {u}^{i}_T$ and $m^{i}_T$ ($i=0,1$) for histological sections in Scenario 1 (second/third row) with two representative images with an immunofluorescence staining of melanoma: with blue (DAPI, cell nuclei), red (CD45, immune cell marker), green (gp100, melanocyte marker) and Scenario 2 (fifth/sixth row) H&E stains of melanoma

Figure 4 depicts the training data and model outputs for the immune cell detection tasks addressed in Scenario 1 and Scenario 2. The training data, which consist of pairs of input RGB image and initial segmentation mask as well as a target pair, are shown along with the corresponding network output in the first row for Scenario 1 and in the fourth row for Scenario 2. The second and third rows show two representative input images of histological sections for Scenario 1 using an immunofluorescence staining of melanoma along with the reconstructed RGB image $\mathbf {u}_T$ and the predicted segmentation mask $m_T$. In the same fashion, two characteristic test input samples using H&E stains are depicted in the fifth and sixth row along with the reconstructed RGB image and the predicted mask. As a result, the predicted segmentation mask and the ground truth mask nearly coincide for synthesized data after a possible threshold. In all cases, the reconstructed RGB image is a significantly denoised version of the input image. Furthermore, the proposed model is capable of generalizing to real image data in Scenario 1, which is verified by three experienced pathologists. In the immune cell classification task in Scenario 2, all immune cell-rich regions are actually detected. The image intensity of the predicted mask correlates with the concentration of immune cells, and thus areas of lower concentration can be masked out by a proper thresholding.

**Fig. 5:**
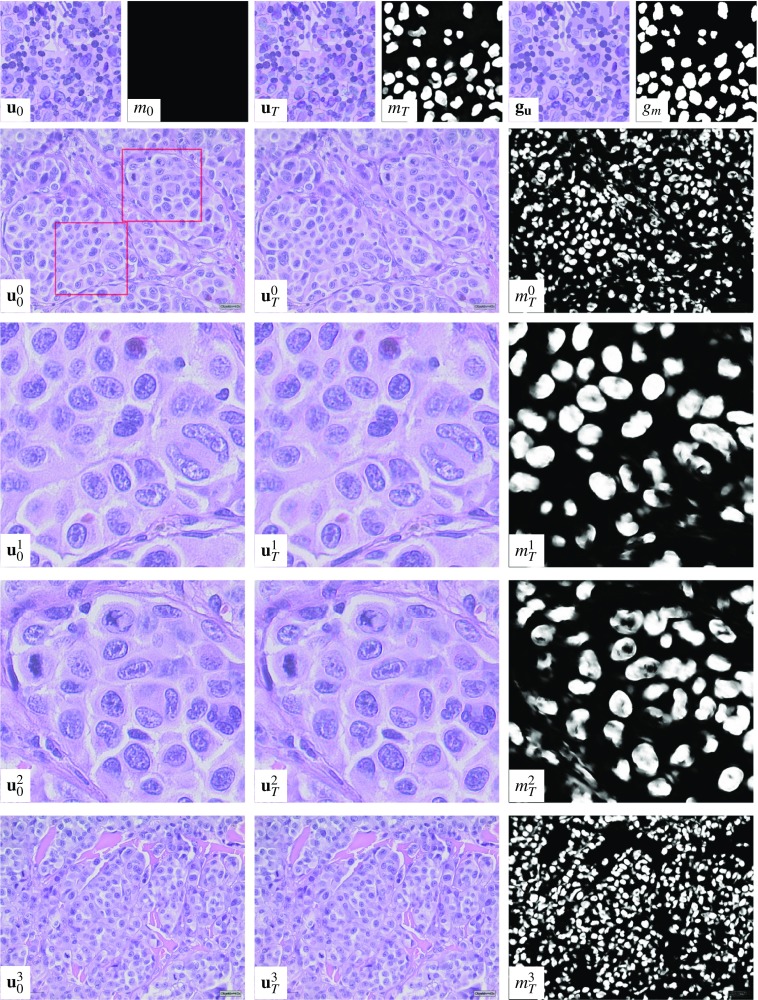
Training data (pairs of image and initial mask, output and ground truth) for Scenario 3 (first row). $\mathbf {u}^{i}_0$, $\mathbf {u}^{i}_T$ and $m^i_T$ ($i=0,1,2,3$) for histological sections in Scenario 3 (second to fifth row) with H&E stains of melanoma. The input images $\mathbf {u}^{1}_0$ and $\mathbf {u}^{2}_0$ are magnified picture details of $\mathbf {u}^{0}_0$. All patches of the tumor nuclei used for generating the synthesized training data are extracted from $\mathbf {u}^{3}_0$

**Fig. 6:**
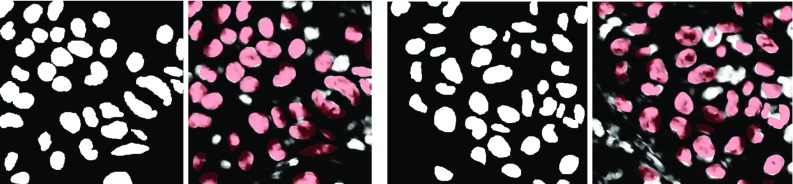
Pairs of annotated masks drawn by a pathologist and overlay of the annotated masks and the computed segmentation masks $m_T$ for the images $\mathbf {u}^1$ (left) and $\mathbf {u}^2$ (right) in Scenario 3

**Fig. 7:**
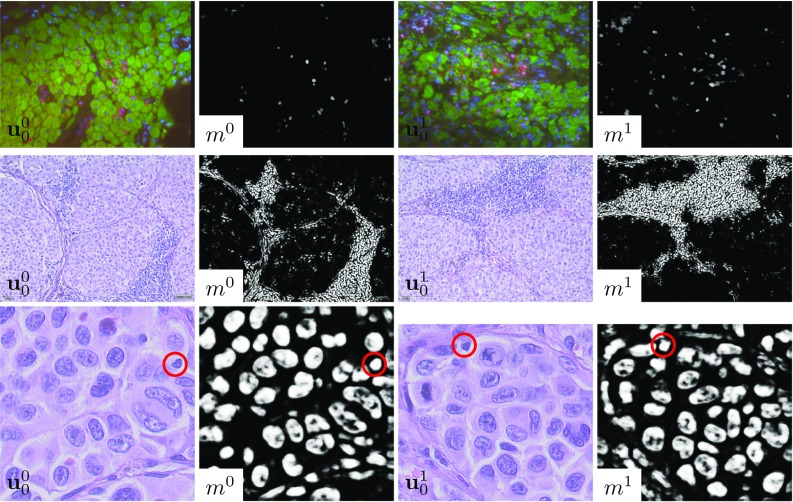
Pairs of images and segmentation masks computed with the U-Net model for Scenario 1 (first row), Scenario 2 (second row) and Scenario 3 (third row). The red circles indicate improperly segmented immune cells

Figure 5 analyzes the performance of the model on tumor cell nuclei detection in Scenario 3. In the first row, an input training sample pair ($\mathbf {u}_0, m_0$) and a target pair ($\mathbf {g}_\mathbf {u}, g_m$) with their corresponding model output pair ($\mathbf {u}_T, m_T$) are shown. The remaining rows depict representative input RGB images using H&E stains of melanoma for Scenario 3 and their corresponding model output pairs. Note that the third and fourth rows highlight patches of the first test sample in the second row. We added the fifth row to show the qualitative performance of the model on images that have been used to extract patches and noise statistics for the stochastic training data generation. As above, the proposed model computes denoised versions of the input images. We highlight that in the predicted segmentation mask all tumor nuclei are actually classified, while at the same time the immune cells are discarded. To quantitatively assess the segmentation masks for Scenario 3, the images $\mathbf {u}^1$ and $\mathbf {u}^2$ were manually annotated as above by three experienced pathologists (cf. the manual segmentations in Fig. 6 along with the overlay of these annotations with the computed segmentation masks $m_T$). The accuracies are $86.20\%$ for $\mathbf {u}^1$, $84.50\%$ for $\mathbf {u}^2$ and $81.05\%$ for $\mathbf {u}^3$, which mainly results from the imprecise masking of the entire cell regions. This proves that the proposed algorithm incorporates both the geometry and the texture of the cells since the restriction to only one attribute would likely result in a poorer accuracy rate.

Finally, we additionally used the standard U-Net architecture [13] as well as variants of this architecture with less parameters instead of the aforementioned variational network. Figure 7 depicts pairs of input images and segmentation masks computed with a U-Net architecture with 3 scales, in which each scale is composed of two convolution layers and subsequent ReLU layers that operate on 32 feature channels. To optimize the model, an Adam optimizer with a learning rate $5\cdot 10^ {-5}$ and 3000 iterations is used. The accuracies are $85.44\%$ for $\mathbf {u}^1$, $82.98\%$ for $\mathbf {u}^2$ and $80.84\%$ for $\mathbf {u}^3$. Although the prediction of the segmentation masks $m_T$ and the ground truth masks $g_m$ only slightly differs for the synthesized data, the resulting trained U-Net models do not generalize to real image data due to overfitting with respect to the training data. In particular, the U-Net models tend to incorrectly segment both the tumor cell nuclei and the immune cells as highlighted by the red circles surrounding some immune cells in Fig. 7. In contrast, the variational network proposed here, which is inspired by classical regularization theory, performs much better when transferred from synthetic training data to real image data.

## Discussion

Cancer histopathology reflects underlying molecular processes and contains phenotypic information that might be predictive for the response to immunotherapy. The convergence of image analysis and deep learning approaches provides new opportunities to explore complex phenotype interactions in cancer tissues and to define novel prognostic tools. In this study, we developed a deep learning approach using variational networks for joint image reconstruction and segmentation on melanoma tissue sections to detect and quantify melanoma cells and their direct interactions with tumor-infiltrating immune cells. Deep learning algorithms have so far not been widely adapted for analysis of histopathological tumor samples. The laborious nature of image annotation and the lack of common databases are limiting the number of training data. Therefore, it is still challenging to evaluate histopathological features by computational approaches based on deep learning. In this work, we were able to analyze the spatial localization and distribution of immune cells within the tumor microenvironment. We used image analysis algorithms to delineate individual cell nuclei and to identify objective nuclear features including shape and texture. Using our proposed method for nuclei detection, we were able to distinguish melanoma cells from surrounding cells in the tumor microenvironment. Our approach can now be applied to clinically annotated melanoma samples to better understand the relationship between nuclear morphology, tumor genetic and epigenetic and clinical outcomes.

In summary, our findings show, in particular, that automated detection of tumor and immune cells in H&E-stained melanoma samples is feasible. This new approach will help to unmask new phenotypic information about tumor immune cell interactions that has a yet unrecognized clinical and scientific value. Future analysis should consider the complex heterogeneity of tumor cell shapes and others cells from the tumor microenvironment like vessels, stroma cells and different types of immune cells. Additionally, multiplex immunohistochemistry that allows for the simultaneous detection of multiple targets of interest in a single tissue section will further extend the phenotypic information and should be implemented in deep learning imaging approaches.
